# Adolescent sexual and reproductive health for all in sub-Saharan Africa: a spotlight on inequalities

**DOI:** 10.1186/s12978-021-01145-4

**Published:** 2021-06-17

**Authors:** Venkatraman Chandra-Mouli, Sarah Neal, Ann-Beth Moller

**Affiliations:** 1 Department of Sexual and Reproductive Health and Research/Human Reproduction Programme, World Health Organization, Geneva, Switzerland; 2 Department of Social Statistics and Demography, University of Southampton, Southampton, UK

The focus of this supplement is on inequalities in the levels and trends of progress on sexual and reproductive health among adolescents in sub-Saharan Africa. Whereas adolescents did not get the attention they deserved in the context of the Millennium Development Goals, there is strong commitment to ensuring that they are not left behind in the context of the Sustainable Development Goals [1]. The need to pay particular attention to their sexual and reproductive health needs was reinforced in the list of key actions for the future implementation of the Programme of Action of the International Conference on Population and Development at the Nairobi Summit [2]. Two recent reports highlight the unequal burden of Sexual and Reproductive Health (SRH) problems in adolescents, and their unequal access to the SRH services. Just-published data suggest that the prevalence of violence against women in relation to intimate partner violence starts early in the lives of girls/young women with nearly one in four of every married/partnered 15–19-year-olds already being subjected to physical and/or sexual violence from an intimate partner at least once, and that the levels of violence in the last 12 months (16%) are higher in this age group [3]. Data released by the Guttmacher Institute stressed that as of 2019, adolescents faced vast unmet needs for sexual and reproductive health services (e.g., 41% of adolescent girls aged 15–19 who wanted to avoid a pregnancy had unmet needs for contraception, whereas the comparable rate in 15–49-year-olds was 24%), and projected that this was likely to worsen in the context of the COVID-19 pandemic’s movement restrictions and service disruptions [4].

There is a pressing need to address the sexual and reproductive health (SRH) of adolescents in sub-Saharan Africa (SSA) because there are glaring inequalities in the levels and trends of key SRH challenges between adolescents in this region when compared with that of adolescents in other parts of the world. For example, adolescents in SSA bear the highest burden of adverse SRH outcomes. One example is HIV infection—the vast majority of new HIV infections among adolescent girls are in East and Southern Africa [5]. Additionally, the majority of the countries with the highest rate of child marriage are in West Africa [6]; and the region as a whole has the highest level of unmet need for modern contraception in adolescents, although there has been impressive progress recently [7].

The papers in this supplement point to the huge inequalities in the levels and trends of health outcomes, harmful practices, health behaviours and in the uptake of SRH services between and within SSA countries, and in the access to preventive and curative health interventions.

- Two key messages emanating from the paper by Kanunura Muhuzuma et al. are that progress has been limited and slow across countries, and that there are enormous disparities between countries [8].
- The paper by Melesse et al. extends this point to within country inequalities, underscoring that disparities in age of sexual debut, early marriage, and early childbearing are persisting and growing across a number of countries [9].
- The paper by Wado et al. notes that while all groups of adolescent girls/young women (and adult women for that matter) are affected by intimate partner violence, those who are from poor families, have limited education and live in rural areas are most affected [10].
- The paper by Cane et al. highlights the disparities in the levels and trends of HIV prevalence between girls/young women and boys/young men, and between rural and urban residents, while also pointing to the considerable progress that has been made in reducing HIV transmission in all groups [11].
- The paper by Kavao Mutua et al. reiterates that the least progress in satisfied needs for family planning over time is among the unmarried and girls/young women from the poorest communities [12].

This body of work powerfully reinforces the point that national averages hide huge disparities. To unmask this by disaggregating data by demographic and socio-economic characteristics from reports of household surveys can provide very useful insights. Going forward it is important that disaggregated data are collected (in future surveys), analyzed, presented and used for decision making.

In moving forward, we see five challenges and opportunities.

Firstly, SRH survey data are increasingly available in the public domain and enable the analysis and presentation of findings disaggregated by age, sex (where appropriate), wealth status, education status and urban–rural residence as well as other attributes such as religion, ethnicity and gender norms/attitudes (covered in other studies, but not in this body of work) [13]. These moves are in the right direction as are moves such as the inclusion of adolescent fertility indicators for those aged 10–14 years and marriage for girls aged below 15 years within the Sustainable Development Goals targets (which till now rely on retrospective studies) [14]. But they need to become universal practice.

Secondly, the papers in this supplement provide important new evidence on the inequalities in SRH outcomes and the determinants of these outcomes including inequalities in access to and uptake of health interventions. However, even more nuanced and granular analysis are needed if those adolescents with the most pressing SRH needs are to be identified. For example, while the SRH situation in urban settings in most SSA countries is better than that in rural areas, the situation in illegal and—often un-serviced—shanty townships that house a large and growing proportion of the population in urban areas is not always so [15]. Given the heterogeneity within urban—but not just urban—environments, further disaggregation is needed. Geospatial survey methods are proving to be useful in identifying geographic “pockets” of poor health outcomes [16]. They will need to be complemented with research studies and administrative data on health, education and social-welfare systems from these areas.

Thirdly, while teasing out the effects of individual factors on health outcomes, harmful practices and health services uptake is important, it is equally as important to understand the synergistic effects of these factors and how they interplay to create and concentrate disadvantage. Intersectionality has rarely been explored in the context of adolescent sexual and reproductive health, yet is vital if we are to have a more holistic understanding of the way in which social systems, power and identity influence outcomes and behaviours. The conceptual framework for action on the social determinants of health [17] provides a powerful equity lens to understand how an intersecting web of factors increases the likelihood of some groups of adolescents/young people being more likely to be ‘exposed’ to a SRH outcome or harmful practice, more likely acquire it, and more likely to suffer negative consequences as a result of acquiring it. For example, a 15 year-old girl in Mali is more likely to be ‘exposed to the risk’ of child marriage than a girl of the same age in Ghana (one in two girls are married before the age of 18 in Mali, whereas one in five are in Ghana). She is more likely to be married as a child if she were growing up in a subsistence farming community in rural Mali than in an urban middle-class family of the country because of community norms and very few other life options. She is also much more likely to suffer health consequences such as very early childbearing with associated complications during pregnancy and childbirth, and rapid repeat pregnancy because of poor access to free quality health care and inability to pay for services provided by private sector providers. She is similarly more likely to be unable to continue with her schooling during her pregnancy or to rejoin a school after childbirth because of the dearth of schools nearby [18, 19].

Fourthly, data on a number of groups of adolescents who may experience greater risks of SRH problems and face greater obstacles in accessing and using SRH interventions, are generally not captured in nationally representative surveys; this includes adolescents who live and work on street, those who are disabled, and those who of diverse sexual orientations and/or gender identities and expressions. While boosting sub samples within general surveys may be appropriate for some groups, others may require specific studies with approaches such as respondent-driven or time-place sampling which are designed to identify and engage hard-to-reach groups [20].

Fifthly, given the nature of the data sources, rapid changes in the demographic and health situation that have occurred because of environmental conditions such as a drought, population movement resulting from insecurity or an epidemic will only become apparent when a new round of the surveys are conducted, which may take years. Stronger Health Information Systems and more regular surveys carried out by Performance Monitoring for Action—and discussed by Kanunura Muhuzuma et al. in this supplement (9)—could play a useful role in filling this gap. Rapid studies could also play a useful complementary role. A case in point is the studies including the Gender and Adolescence—Global Evidence Alliance that have been carried out by a number of organization that point to the effects of the closures, service disruptions and movement restrictions caused by the COVID-19 pandemic [21].

Finally, data is only useful if it is used to inform policies and programmes. In many places, the growing body of abundant epidemiologic information and findings from social and behavioural studies, do not feed into policy formulation, programme design or—as George et al. have shown—in investment case development [22]. This must change. Methods and tools to assess which groups of adolescents are being left behind in programmes and projects and how best to include them are available [23, 24]. The approaches recommended by WHO and Population Council have been tried and tested in projects and are now being applied in large scale programmes particularly in the context of HIV prevention [25]. To meet the needs of the many adolescents who have been—and are being left behind—these  approaches must become universal practice.

## Data Availability

Not applicable.
